# Management of Synovial Osteochondromatosis of the Distal Radioulnar Joint with Imaging Features Consistent with Malignancy

**DOI:** 10.1155/2013/589631

**Published:** 2013-09-19

**Authors:** Colin W. McInnes, Thomas J. Goetz

**Affiliations:** ^1^Section of Plastic Surgery, University of Manitoba, Winnipeg, MB, Canada R3A 1R9; ^2^Department of Orthopaedics, University of British Columbia, Vancouver, BC, Canada V6Z 2A5

## Abstract

Synovial osteochondromatosis of the distal radioulnar joint is a rare entity with only 14 cases reported in the literature. Malignant transformation of synovial osteochondromatosis is the most worrisome complication of the disease. It has been described in joints such as the hip and knee but never for the distal radioulnar joint. We report a case of synovial osteochondromatosis of the distal radioulnar joint which presented with radiographic features which were worrisome for malignant transformation and required a comprehensive preoperative workup. Discussed are the preoperative management, surgical treatment, and a literature review of this rare disease.

## 1. Introduction

Synovial osteochondromatosis of the wrist is a rare, progressive disorder characterized by benign hyperplastic metaplasia of the subsynovial cells resulting in cartilaginous nodules which often shed loose bodies into the synovial cavity [1]. Common clinical symptoms are nonspecific and include pain, swelling, and restricted range of motion that may progress slowly [2]. Most patients with synovial osteochondromatosis of the wrist present in the 3rd or 4th decade of life, and men are more likely to present than women [3]. Treatment involves the surgical excision of the tumor and usually has a satisfactory outcome.

There have been fewer than 15 previous reports of synovial osteochondromatosis of the distal radioulnar joint [4]. Herein, we report an additional case which presented with radiographic features worrisome for a soft tissue sarcoma. A literature review of the clinical, radiologic, and therapeutic modalities of synovial osteochondromatosis of the wrist is discussed.

## 2. Case History

A 28-year-old, right hand dominant female presented with a 3-year history of an enlarging mass in her left distal forearm which had become increasingly painful. Her wrist was initially sore only while typing but later also became painful at rest. The patient did not experience any systemic symptoms such as weight loss or night sweats and had no history of sarcoma or other tumors. She was otherwise healthy with a benign medical history, no previous surgeries, and no prior injuries to her left hand or wrist.

On physical examination, she had a palpable mass, approximately 3 cm × 3 cm in diameter, located over the dorsal and volar aspects of the left distal wrist. Flexor and extensor tendons appeared to be superficial to the mass, and she had full range of motion of the wrist with the exception of a loss of 20 degrees of pronation. Neurovascular status of the left hand was normal. 

**Figure 1:**
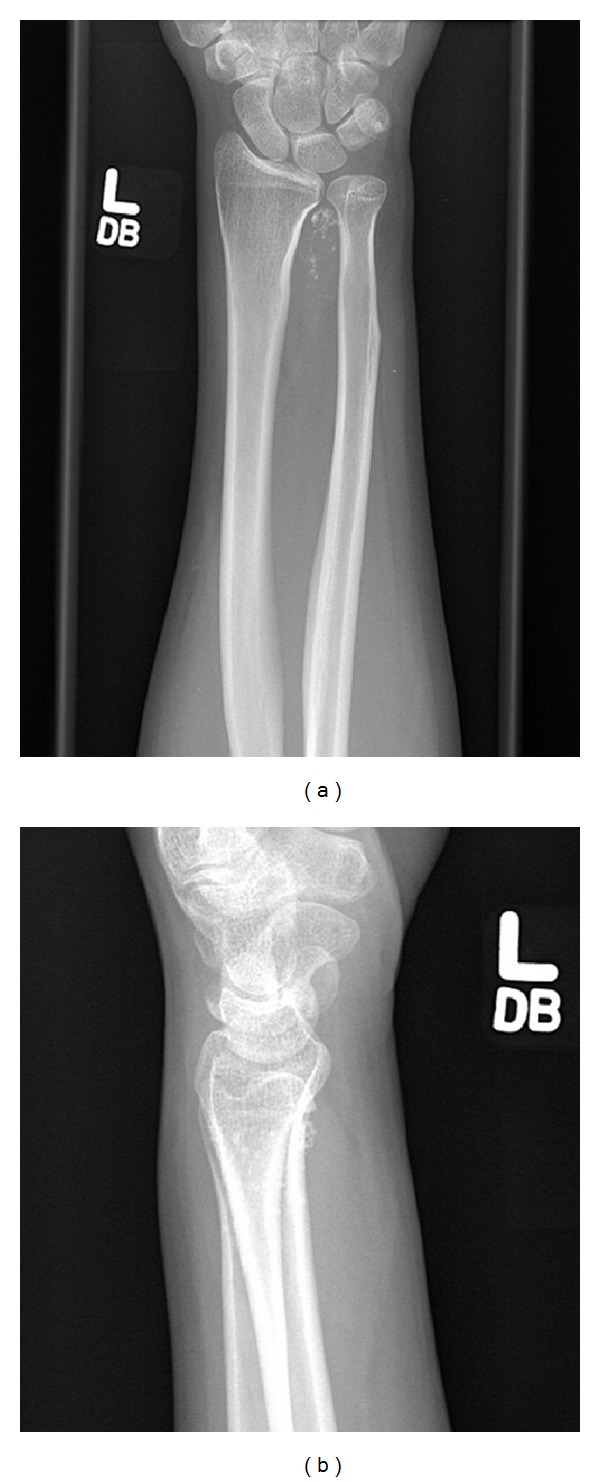
(a) and (b) PA and lateral X-rays of the left forearm and wrist showing calcified nodules consistent with synovial chondromatosis. Superficial cortical scalloping of the distal radius and ulna is noted.

**Figure 2:**
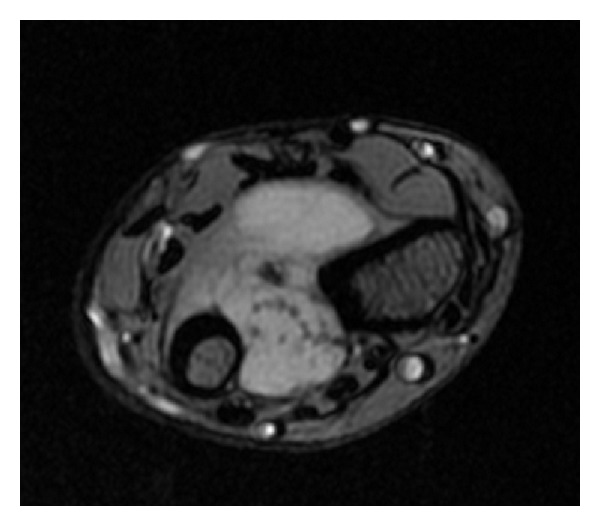
MRI of the left distal radioulnar joint showing calcified nodules consistent with synovial osteochondromatosis.

Radiographs showed a mass with dystrophic, amorphous calcifications centered in the interosseous membrane, just proximal to the distal radioulnar joint (Figure 1). There was superficial scalloping of the adjacent radial and ulnar cortices but no evidence of periosteal thickening or destruction. An MRI showed a 2.8 cm × 2.3 cm × 3.2 cm mass located in the left interosseous membrane that encroached the distal radioulnar joint and the dorsal radiocarpal joint (Figure 2). The mass did not appear to invade the adjacent muscle compartments, tendons, or neurovascular bundles. The mass did not enhance on T1 sequence but had high signal intensity on proton density sequence and demonstrated irregular peripheral and nodular enhancement. The features on imaging were considered consistent with malignant fibrous histiocytoma or fibrosarcoma.

Due to concern of a potential malignant process, a CT guided biopsy of the mass and a CT chest were ordered to help rule out sarcoma. Histopathology of the biopsy showed fragments of a well-differentiated hyaline cartilage with focal calcifications, and a preliminary diagnosis of synovial osteochondromatosis was made.

**Figure 3:**
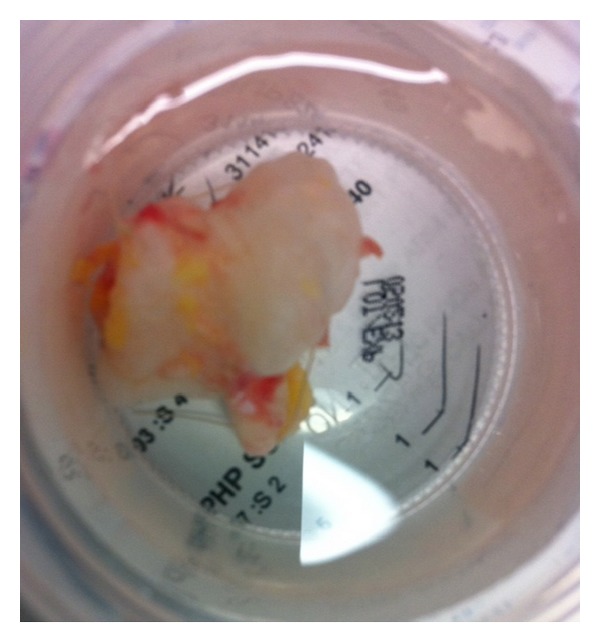
Gross pathological specimen of resected tumor showing dumbbell configuration.

Surgical indication for excisional biopsy was made as the patient described pain, loss of motion, and concern about the mass. Both volar and dorsal incisions of the forearm were required to excise the mass as it was located on both sides on the interosseous membrane in a dumbbell-shaped configuration (Figure 3). The most distal aspect of the mass entered the distal radioulnar joint. The triangular fibrocartilage complex (TFCC) and the dorsal capsule of the distal radioulnar joint were preserved. The distal aspect of the volar radioulnar joint capsule was excised. There were no intraoperative complications. 

Consistent with the initial biopsy, histological analysis of the excised specimen showed that the majority of the tumor was encapsulated and demonstrated hyaline cartilage with focal calcifications. A final diagnosis of synovial osteochondromatosis was made.

The patient has done well postoperatively, with no evidence of recurrence and a return of full range of motion of her left wrist 2 years postoperatively. 

## 3. Discussion 

Synovial osteochondromatosis is a benign condition of unknown etiology, whereby a metaplasia of the synovial membrane results in an accumulation of intra-articular cartilaginous nodules. Common clinical features include mild pain, swelling, and stiffness of the affected joint. Locking and clicking due to loose bodies have also been reported [5]. It has been classified into 3 phases: the first shows active intrasynovial disease without loose bodies, the second shows intrasynovial disease with loose bodies, and the third shows multiple osteochondral bodies but no intrasynovial disease [1]. It is most common in the larger joints such as the knee, hip, shoulder, and elbow and is relatively uncommon in the hand and wrist. Synovial osteochondromatosis of the distal radioulnar joint is most commonly found in males in the 2nd through 4th decades of life [4]. Prior injury of the wrist does not appear to increase the likelihood of developing this condition in the distal radioulnar joint [3]. 

The differential diagnosis for synovial osteochondromatosis in the wrist is broad and includes benign and malignant tumors. The latter includes synovial chondrosarcoma and malignant fibrous histiocytoma, and the former includes synovial osteochondromatosis, rheumatoid or osteoarthritis, and chondrocalcinosis [6]. A tissue biopsy with histological analysis remains the gold standard for diagnosis, although unequivocal histological diagnosis can be challenging, and correlation with clinical and radiological features is often recommended to aid in differentiating benign and malignant entities [6, 7]. There have been rare reports of malignant transformation of synovial osteochondromatosis, and recurrence following excision should alert the clinician to a possible malignant process [6–8].

Clinicians must rely primarily on clinical exam and imaging when diagnosing synovial osteochondromatosis preoperatively. While the radiographic features of this condition are variable depending on the maturity of the tumor, abnormalities can be appreciated in most cases. Initially, the tumor may present with an effusion and noncalcified loose bodies, which may not be visible on radiographs but can usually be visualized on CT or MRI [9]. As the mass matures, features such as superficial bone erosions and calcified loose bodies are often apparent [8]. In this case, scalloping of the adjacent radial and ulnar cortices was seen which, despite being common in synovial osteochondromatosis, is also a finding consistent with chondrosarcoma [6, 8]. While it is generally acceptable to excise synovial osteochondromatosis based on clinical and radiographic evidence suggestive of the disease, in this case, it was necessary to obtain a tissue sample in order to rule out sarcoma prior to the excision. 

The current standard for the treatment of synovial osteochondromatosis involves the removal of any nodules and loose bodies from the synovial cavity and excision of diseased synovium [4, 10]. Postoperative recurrence has been reported at 17% for synovial osteochondromatosis of the wrist, and patients tend to do well clinically following excision of the mass [4]. In cases of recurrence, additional surgery is usually indicated.
